# Supplementary material for: Identification of photoperiod-induced specific miRNAs in the adrenal glands of Sunite sheep (Ovis aries)
Source: Front Vet Sci. 2022 Jul 22;9:888207. doi: 10.3389/fvets.2022.888207 (PMC9354845; doi:10.3389/fvets.2022.888207)
Supplement: Supplementary file 1 [file Data_Sheet_1.ZIP › Supplementary file/miRDeep2 file.zip!/miRDeep2 file/Know_Structure/SP42A1/oar-mir-374b.pdf]

miRBase precursor	: oar-mir-374b
Total read count	: 22867
oar-miR-374b read count	: 22374
remaining reads	: 493

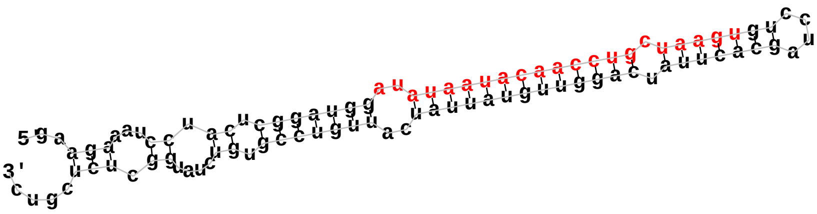

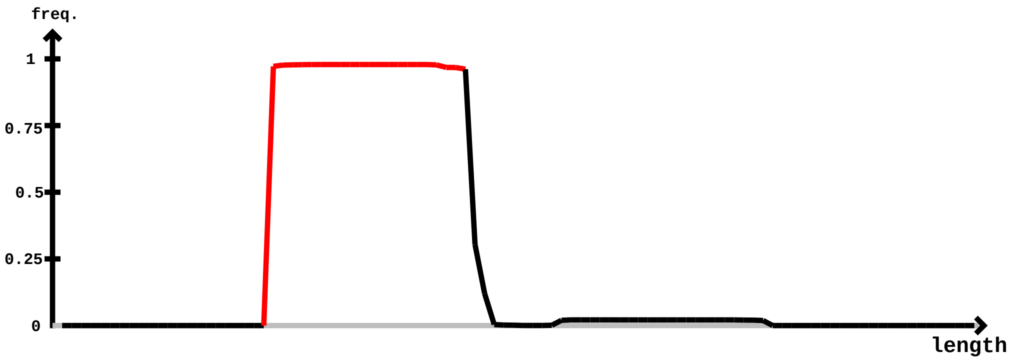


```
                      oar-miR-374b
5'- gaagaaauccuacucggauggauauaauacaaccugcuaaguguccuagcacuuaucagguuguauuaucauuguccgugucuauggcucucguc  -3'   exp
    ((((..((...((.(((((((..(((((((((((((.(((((((....))))))).)))))))))))))..))))))).))..))..)).))...    reads   mm     sample
    .....................auauGauacaaccugcu.........................................................    1       1      seq
    .....................auauaauacaaccugcu.........................................................    32      0      seq
    .....................auauaauacaaccugcua........................................................    213     0      seq
    .....................auauaauacaaccugcuaa.......................................................    10      0      seq
    .....................auauaauacaaccugcuaag......................................................    143     0      seq
    .....................auauaauacaaccugcuaagu.....................................................    14998   0      seq
    .....................auauaauacaaccugcuaagug....................................................    4200    0      seq
    .....................auauaauacaaccugcuaagugu...................................................    2587    0      seq
    .....................auauaauacaaccugcuaaguguc..................................................    32      0      seq
    .....................auauaauacaaccugcuaagugucc.................................................    6       0      seq
    .....................auauaauacaaccugcuaaguguccu................................................    3       0      seq
    .....................auauaauacaaccugcuaaguguccuagc............................................    1       0      seq
    ......................uauaauacaaccugcuaagu.....................................................    23      0      seq
    ......................uauaauacaaccugcuaagug....................................................    5       0      seq
    ......................uauaauacaaccugcuaagugu...................................................    69      0      seq
    ......................uauaauacaaccugcuaaguguc..................................................    9       0      seq
    ......................uauaauacaaccugcuaagugucc.................................................    1       0      seq
    ......................uauaauacaaccugcuaaguguccu................................................    1       0      seq
    .......................auaauacaaccugcuaagu.....................................................    8       0      seq
    .......................auaauacaaccugcuaagug....................................................    3       0      seq
    .......................auaauacaaccugcuaagugu...................................................    2       0      seq
    .......................auaauacaaccugcuaaguguc..................................................    2       0      seq
    .......................auaauacaaccugcuaaguguccu................................................    3       0      seq
    ........................uaauacaaccugcuaaguguc..................................................    1       0      seq
    ........................uaauacaaccugcuaagugucc.................................................    2       0      seq
    ........................uaauacaaccugcuaaguguccu................................................    14      0      seq
    ........................uaauacaaccugcuaaguguccua...............................................    2       0      seq
    ........................uaauacaaccugcuaaguguccuag..............................................    1       0      seq
    ........................uaauacaaccugcuaaguguccuagcacuu.........................................    1       0      seq
    ........................uaauacaaccugcuaaguguccuagcacuuauc......................................    1       0      seq
    .........................aauacaaccugcuaagugucc.................................................    2       0      seq
    .........................aauacaaccugcuaaguguccu................................................    3       0      seq
    ..........................auacaaccugcuaaguguccu................................................    1       0      seq
    ..........................auacaaccugcuaaguguccuagc............................................    2       0      seq
    .................................................cacuuaucagguuguauuau..........................    1       0      seq
```

oar-miR-374b

```
gaagaaauccuacucggauggauauaaauacaaccugcuaagucuccuagcacuuaucagguuguauuaucauuguccgugucuauggcucucguc
..................................................cacuuaucagguuguauuauc.........................    1     0     seq
...................................................acuuaucagguuguauuau..........................    1     0     seq
...................................................acuuaucagguuguauuauc.........................    7     0     seq
...................................................acuuaucagguuguauuauA.........................    2     1     seq
...................................................acuuaucagguuguauuauca........................    2     0     seq
...................................................acuuaucagguuguauuaucau.......................    10    0     seq
...................................................acuuaucagguuguauuaucauu......................    1     0     seq
....................................................cuuaucagguuguauuauc.........................    4     0     seq
....................................................cuuaucagguuguauuauca........................    3     0     seq
....................................................cuuaucagguuguauuauAau.......................    2     1     seq
....................................................cuuaucagguuguauuaucau.......................    17    0     seq
....................................................cuuaucagguuguauuaucauu......................    395   0     seq
.....................................................uuaucagguuguauuaucauu......................    39    0     seq
```
